# Meningeal Melanocytes in the Mouse: Distribution and Dependence on *Mitf*

**DOI:** 10.3389/fnana.2015.00149

**Published:** 2015-11-25

**Authors:** Stefán A. H. Gudjohnsen, Diahann A. M. Atacho, Franck Gesbert, Graca Raposo, Ilse Hurbain, Lionel Larue, Eirikur Steingrimsson, Petur Henry Petersen

**Affiliations:** ^1^Faculty of Medicine, Department of Anatomy, Biomedical Center, University of IcelandReykjavik, Iceland; ^2^Faculty of Medicine, Department of Biochemistry and Molecular Biology, Biomedical Center, University of IcelandReykjavik, Iceland; ^3^Institut Curie, PSL Research University, INSERM U1021, Normal and Pathological Development of MelanocytesOrsay, France; ^4^Université Paris-Sud, Université Paris-Saclay, CNRS UMR 3347Orsay, France; ^5^Equipe Labellisée Ligue Contre le CancerOrsay, France; ^6^Institut Curie, PSL Research UniversityParis, France; ^7^CNRS UMR144, Structure and Membrane Compartments, and Cell and Tissue Imaging Facility (PICT-IBiSA)Paris, France

**Keywords:** melanocytes, meninges, mouse, *Mitf*, meningeal melanoma

## Abstract

**Summary**: Melanocytes are pigment producing cells derived from the neural crest. They are primarily found in the skin and hair follicles, but can also be found in other tissues including the eye, ear and heart. Here, we describe the distribution of pigmented cells in C57BL/6J mouse meninges, the membranes that envelope the brain. These cells contain melanosomes of all four stages of development and they depend on Microphthalmia associated transcription factor (*MITF*), the master regulator of melanocyte development, suggesting that they are bona-fide melanocytes. The location of these pigmented cells is consistent with the location of meningeal melanomas in humans and animal models.

**Significance**: Here, we document and define pigmented cells in the meninges of the mouse brain and confirm that they are melanocytes. This is important for understanding the role of this cell type and for understanding primary meningeal melanoma, a rare disease that likely arises from normal meningeal melanocytes.

## Introduction

Melanocytes are neural crest derived cells that are primarily found in the skin and hair follicles in humans and are largely restricted to the hair follicles in mice. Their function in pigmentation and ultra-violet (UV) response has been well characterized. Melanocytes are also present in other organs such as the eye and inner ear (Colombo et al., 2011). In these tissues, the melanocytes are known to depend on the Microphthalmia associated transcription factor (*Mitf*). Mice which carry a mutation in the *Mitf* gene have white coat color due to lack of hair follicle melanocytes (Hodgkinson et al., 1993), they exhibit microphthalmia due to defects in the retinal pigment epithelial cells of the eye (Steingrimsson et al., 2004) and they are deaf due to the pivotal role of melanocytes in the inner ear (Ni et al., 2013).

Melanocytes have been shown to exist in other regions of the body including in the mouse heart (Yajima and Larue, 2008). Pigmented cells have also been described in the meninges of the mouse, rat, cat and humans (Barden and Levine, 1983; Goldgeier et al., 1984; Morse and Cova, 1984; Fetissov et al., 1999). The role of melanocytes in the heart and in the meninges is currently unknown. Also, the histological distribution of meningeal melanocytes and their dependence on *MITF*, the master regulator of melanocyte development, has not been established. Here, we describe the distribution of pigmented cells in the meninges of mice and demonstrate that they have characteristics of melanocytes as they contain melanosomes and depend on *Mitf*.

## Materials and Methods

### Mice

C57BL/6J, C57BL/6-*Mitf^vga9^* and C57BL/6-*Tyr^c−2J^* mice were bred and housed in an animal facility at the University of Iceland. The use of laboratory animals was approved by the Icelandic Food and Veterinary Authority (permit ID: 2013-03-01) with all maintenance and handling conducted in accordance with Icelandic law on animal welfare. C57BL/6 mice were also housed in pathogen-free conditions at Institut Curie, conforming to French and European Union legislation.

### Examination of Pigment Cells

**Figure 1:**
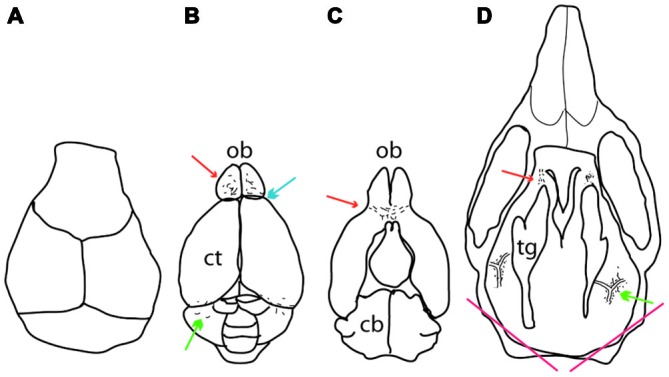
**Location of meningeal pigment cells as they appear during dissection. (A)** Dorsal view of the calvarium after it has been removed, revealing the brain. **(B)** A dorsal view of the brain after it has been removed from the skull. Melanocytes can be found on top of the olfactory bulb anteriorly (red arrow), between the olfactory bulb and the cortex (blue arrow) and between the cerebellum and cortex posteriorly (green arrow). **(C)** The ventral aspect of the brain showing pigment cells underneath the olfactory bulb (red arrow). **(D)** Dorsal view of the cranial floor of the skull after the brain has been removed. Pigmented cells can be seen posteriorly surrounding the pterygopalatine artery where it branches off into the middle meningeal artery lateral to the trigeminal ganglion on either side (green arrow). Anteriorly, pigment cells can be found around the optic and trigeminal nerves where they exit the skull (red arrow). At the bottom of the image are two red lines which indicate where the skull was cut to determine whether inner ear melanocytes were present or not. In all panels, up is anterior and down posterior. Ct, cortex; ob, olfactory bulb; cb, cerebellum; tg, trigeminal ganglion.

To examine the meningeal pigment cells, the mice were sacrificed and the calvarium carefully removed (Figure 1A). After the removal of the calvarium, the brain and olfactory bulb were exposed at which point pigmented cells could be observed on top of the olfactory bulb and between the cerebellum and cortex. Next, the brain was carefully removed and pigmented cells observed underneath the olfactory bulb and between the olfactory bulb and cortex. Finally the cranial floor was examined. Inner ear melanocytes were examined by sectioning through the skull as indicated in Figure 1.

### Histochemical Staining

For staining of sections of the pterygopalatine artery, whole heads were decalcified, fixed and embedded in paraffin. They were then sectioned and stained either using the standard hematoxylin and eosin stain or the Masson-Hamperl silver stain for melanocytes (Grimelius et al., 1994). A section from a human primary melanoma specimen was used as a positive control for pigmented cells containing melanin.

### Electron Microscopy

For transmission electron microscopy (TEM), samples were obtained by careful dissection of the area between the olfactory bulb and the cortex and from the pterygopalatine artery of C57BL/6J mice and fixed overnight in 2.5% (v/v) glutaraldehyde in PBS. The samples were then embedded in resin, trimmed, rough sectioned and stained with toluidine blue. After appropriate sections had been found, thin-sectioned samples of 60–70 nm were post stained with 4% (v/v) aqueous uranyl acetate for 10 min and lead citrate for 1 min. Electron micrographs were acquired on a Tecnai Spirit G2 electron microscope (FEI, Eindhoven, Netherlands) equipped with a 4 k CCD camera (Quemesa, Olympus, Münster, Germany).

### RT-PCR

For RT-PCR, C57BL/6J, C57BL/6J-*Mitf^mi−vga9^/Mitf^mi−vga9^*, C57BL/6J-*Mitf^mi−vga9^*/+ and C57BL/6J-Tyr^c-2J/^Tyr^c-2J^ mice were sacrificed and the meninges obtained by carefully removing the brain and subsequently peeling the meninges from the brain around the olfactory bulb. Meninges from three mice per genotype were pooled, and RNA isolated using a phenol-chloroform extraction method (Chomczynski and Sacchi, 1987). Similarly, RNA was isolated from the B16-F1 mouse melanoma cell line after culture in DMEM medium containing 1% Penicillin-streptomycin and 10% Fetal Bovine Serum under standard conditions. cDNA was synthesized using 0.3 ng of total RNA using SuperscriptII RT from Invitrogen (Carlsbad, USA). RT-PCR was performed using HotStart Taq Polymerase from Qiagen (Hilden, Germany). The following primer pairs were used: *Mitf*-M Forward: 5^′^-GGAAATGCTAGAATACAGTCACTACC-3^′^, *MITF*-M Reverse: 5^′^-CATGCACGACGCTCGAGAGTGC-3^′^, Pmel Forward: 5^′^-ACCTGGGGAAAATACTGGCA-3^′^, Pmel Reverse: 5^′^-AGGAAGTGCTTGGTCTCTCC-3^′^, beta-actin Forward: 5^′^-CACAGCTGAGAGGGAAATCG-3^′^, beta-actin Reverse: 5^′^-GATCTTGATCTTCATGGTGC-3^′^. Bands were observed at the predicted band size of 337 bp for *Mitf*-M, 236 bp for Pmel and 382 bp for beta-actin.

## Results

### Distribution of Pigmented Cells in the Meninges

**Figure 2:**
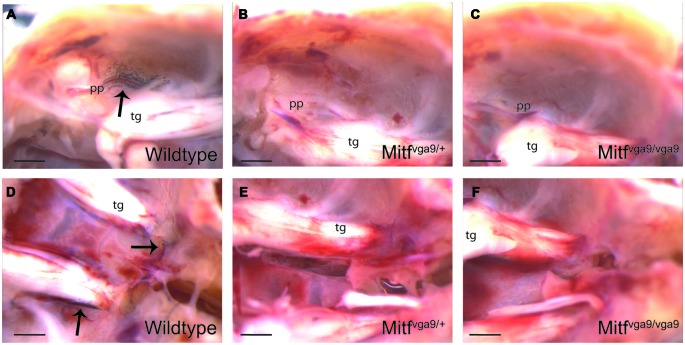
**Pigmented cells in the cranial floor. (A–C)** The pterygopalatine artery where it branches off into the middle meningeal artery in wild type, *Mitf^vga9^/+* and *Mitf^vga9^/Mitf^vga9^* mice. **(D–F)** The trigeminal nerve and the exit point for the optic nerve and the optic nerve in wild type, *Mitf^vga9^*/+ and *Mitf^vga9^/Mitf^vga9^* mice. Arrows point to the pigmented cells. In all panels, left is posterior and right anterior. Tg, trigeminal ganglion; pp, pterygopalatine artery. Scale bar represents 1 mm.

**Figure 3:**
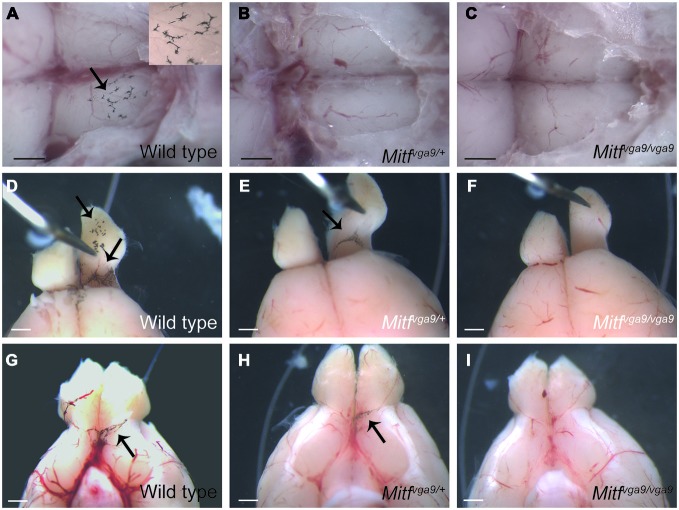
**Pigmented cells around the olfactory bulb. (A–C)** A dorsal view of the olfactory bulb in wild type, *Mitf^mi−vga9^/+* and *Mitf^mi−vga9^/Mitf^mi−vga9^* mice. The insert in **(A)** shows a greater magnification of a portion of the image. Anterior is to the right. **(D–F)** Dorsal view of the area between the olfactory bulb and cortex in wild type, *Mitf^mi−vga9^*/+ and *Mitf^mi−vga9^/Mitf^mi−vga9^* mice. **(G–I)** A ventral view of the brain and in particular the olfactory bulb in wild type, *Mitf^mi−vga9^*/+ and *Mitf^mi−vga9^/Mitf^mi−vga9^* mice. Arrows point towards pigment cells. Anterior is up in **(D–I)**. Scale bars represent 1mm.

To determine the distribution of pigmented cells in the meninges of mice, 45 wild type C57BL/6J mice of both sexes, ranging in age from 0.5–19 months, were examined. Dendritic, pigmented cells were found in the following locations: (i) Between the olfactory bulb and the cortex, occasionally extending into the anterior interhemispheric area (blue arrow in Figure 1B); (ii) on top of the olfactory bulb (red arrow in Figure 1B); (iii) between the cerebellum and the cortex (green arrow in Figure 1B); (iv) under the olfactory bulb (red arrow in Figure 1C); (v) around the pterygopalatine and middle meningeal artery at their junction lateral to the trigeminal ganglion (green arrow in Figure 1D); and (vi) around the optic and trigeminal nerves anteriorly (red arrow in Figure 1D). The pigmented cells in the cranial floor meninges are mainly localized around the pterygopalatine artery where it branches off into the middle meningeal artery and at the exit points of the optic and trigeminal nerves from the skull (Figures 2A,D). The pigmented cells in the meninges surround the olfactory bulb (Figures 3A,D,G), and are found between the cerebellum and cortex. These pigmented cells were also observed in C57BL/6J mice housed in Orsay, France.

Pigmented cells were most abundant around the olfactory bulb and surrounding the pterygopalatine artery in the cranial floor (Figures 2, 2, 3). However, we noticed that the presence of pigmented cells in the different regions varied between animals of the isogenic C57BL/6J strain. In order to determine the prevalence of pigmented cells in the different areas, the number of mice containing pigmented cells in the different regions was determined. Table 1 shows the prevalence of pigment containing cells in the cranial floor and inner ear. In wild type mice, the inner ear and left and right optic nerves nearly always contain pigmented cells. They are common but less frequently observed around the left and right blood vessels (Table 1). Table 2 shows the distribution of pigmented cells around the brain. In wild type mice the ventral olfactory bulb and the region between the olfactory bulb and cortex frequently have pigmented cells but they are less frequently observed on the dorsal olfactory bulb and in the region between the cerebellum and cortex (Table 2). The pigmented cells were sometimes present only on the left side of the animal and sometimes only on the right side. In order to quantify this we analyzed their bilateral distribution. Table 3 shows whether the cells are unilaterally distributed, bilaterally distributed or absent from each area. These cells are bilaterally present in the inner ear, optic nerve and ventral olfactory bulb but are either not present or unilaterally present on either the left or right side around the blood vessel, between the cerebellum and cortex and on the dorsal side of the olfactory bulb.

**Table 1:** **Distribution of meningeal pigment cells in the cranial floor and inner ear of mice**.

Genotype	Left inner ear	Left blood vessel	Left optic nerve	Right optic nerve	Right blood vessel	Right inner ear
Wild type (*n* = 45)	44 (98%)	27 (60%)	43 (96%)	40 (89%)	25 (56%)	43 (96%)
*Mitf*^vga9^/+ (*n* = 25)	23 (92%)	0 (0%)	0 (0%)	2 (8%)	0 (0%)	24 (96%)
*Mitf*^vga9^/*Mitf*^vga9^ (*n* = 25)	0 (0%)	0 (0%)	0 (0%)	0 (0%)	0 (0%)	0 (0%)

**Table 2:** **The distribution of pigment cells in the meninges around the brain and olfactory bulb**.

	Between the cerebellum and cortex		Dorsal olfactory bulb		Ventral olfactory bulb		Between the olfactory
Genotype	Left	Right	Left	Right	Left	Right	Bulb and the cortex
Wild type (*n* = 28)	4 (14%)	7 (25%)	8 (29%)	10 (36%)	21 (75%)	18 (64%)	21 (75%)
*Mitf*^vga9^/+ (*n* = 12)	0 (0%)	0 (0%)	0 (0%)	0 (0%)	5 (42%)	2 (17%)	5 (42%)
*Mitf*^vga9^/*Mitf*^vga9^ (*n* = 12)	0 (0%)	0 (0%)	0 (0%)	0 (0%)	0 (0%)	0 (0%)	0 (0%)

**Table 3:** **The distribution of pigment cells: unilateral vs. bilateral in wild type**.

Area	Not present	Unilateral left	Unilateral right	Bilateral
Inner ear (*n* = 45)	0 (0%)	1 (2%)	2 (5%)	42 (93%)
Blood vessel (*n* = 45)	10 (22%)	10 (22%)	8 (18%)	17 (38%)
Optic nerve (*n* = 45)	2 (5%)	3 (7%)	0 (0%)	40 (88%)
Between cerebellum and cortex (*n* = 28)	19 (68%)	2 (7%)	5 (18%)	2 (7%)
Dorsal olfactory bulb (*n* = 28)	15 (53%)	3 (11%)	5 (18%)	5 (18%)
Ventral olfactory bulb (*n* = 28)	6 (21%)	4 (14%)	1 (4%)	17 (61%)

### Distribution of Pigment Cells Varies with Age

To determine whether age had an effect on the distribution of these cells, the wild type C57BL/6J mice were divided into three groups, each with 15 mice: young (under 2 months), adult (between 2 and 8 months) and old (older than 8 months old). The presence and distribution of pigmented cells was determined in each area for each age group (Tables 4, 5). Pigmented cells were nearly always observed in the inner ear. Fewer old mice had pigmented cells in the optic nerve area than were seen in young or adult mice, suggesting that these cells disappear from these areas with age (Table 4). The prevalence of pigmented cells around the meningeal blood vessels did not change with age. Interestingly, we observed that pigmented cells were more common on top of the olfactory bulb in young mice (45.5% of the mice show pigmented cells in this area—average of left and right side) than in older mice (19%). In contrast, pigment cells were more prevalent on the ventral olfactory bulb in old mice (94%) than in young mice (59.5%).

**Table 4:** **The distribution of pigmented cells in the cranial floor and in the inner ear by age group**.

Age	Left inner ear	Left blood vessel	Left optic nerve	Right optic nerve	Right blood vessel	Right inner ear
Young (*n* = 15)	15 (100%)	8 (53%)	15 (100%)	15 (100%)	9 (60%)	15 (100%)
Adult (*n* = 15)	14 (93%)	9 (60%)	15 (100%)	14 (93%)	7 (47%)	13 (87%)
Old (*n* = 15)	15 (100%)	10 (67%)	13 (87%)	11 (73%)	9 (60%)	15 (100%)

**Table 5:** **The distribution of pigmented cells in the meninges surrounding the brain and olfactory bulb**.

	Between the cerebellum and cortex		Dorsal olfactory bulb		Ventral olfactory bulb		Between the olfactory
Age	Left	Right	Left	Right	Left	Right	Bulb and cortex
Young (*n* = 11)	0 (0%)	2 (18%)	4 (36%)	6 (55%)	7 (64%)	6 (55%)	9 (82%)
Adult (*n* = 9)	0 (0%)	3 (33%)	2 (22%)	2 (22%)	7 (78%)	4 (44%)	5 (56%)
Old (*n* = 8)	4 (50%)	2 (25%)	1 (13%)	2 (25%)	7 (88%)	8 (100%)	7 (88%)

### Pigmented Cells in the Meninges Depend on *Mitf*

To determine whether the pigmented cells in the meninges depend on *Mitf* expression, their presence and distribution was determined in C57BL/6-*Mitf^mi−vga9^* homo- and heterozygous mice. *Mitf^mi−vga9^*/+ mice contain melanocytes in the inner ear and meningeal pigment cells below the olfactory bulb and between the olfactory bulb and the cortex (Figures 2B,E, 3B,E,H; Tables 1, 1, 2). However, the pigmented cells were missing from all other areas of the brain examined. Importantly, in the homozygous C57BL/6-*Mitf^mi−vga9^/Mitf^mi−vga9^* mice, no pigmented cells were present, demonstrating that the inner ear and meningeal pigment cells depend on *Mitf* (Figures 2C,F, 3C,F,I; Tables 1, 1, 2). Pigmented cells were not observed in C57BL/6J-albino mice (C57BL/6J-*Tyr^c-2J^/Tyr^c-2J^*) which do not produce melanin.

**Figure 4:**
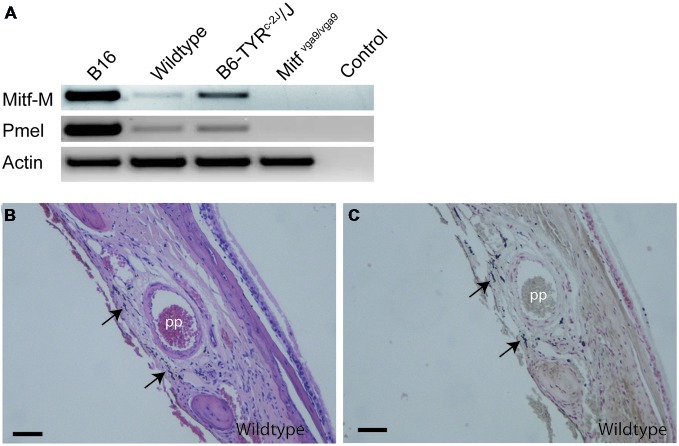
**Location of melanocytes around the pterygopalatine artery. (A)** RT-PCR performed on cDNA obtained from B16 mouse melanoma cells as positive control, olfactory bulb meninges from C57Bl/J6, B6Tyr^c-2J^/Tyr^c-2J^ and *Mitf^mi−vga9^/Mitf^mi−vga9^* mice and no-cDNA negative control. Primers used were for *Mitf-M*, *Pmel*, and *Actin*. **(B)** A coronal section of the pterygopalatine artery stained with H&E. **(C)** An image of an adjoining section stained with Masson-Hamperl silverstain to detect the presence of melanin. Pp, pterygopalatine artery. Scale bars represent 100 μm.

To determine whether the *M-Mitf* (encoding a melanocyte specific isoform of *Mitf*) and *Pmel* (encoding a type I transmembrane glycoprotein) genes, two genetic markers for melanocytes, are expressed in mouse meninges, RT-PCR was performed on meninges isolated from C57BL/6J, C57BL/6J-*Tyr^c-2J^/Tyr^c-2J^* and C57BL/6J-*Mitf^mi−vga9^/Mitf^mi−vga9^* mice, using B16 mouse melanoma cells as a positive control. Since melanin has been shown to inhibit RNA and cDNA preparations (Eckhart et al., 2000), C57BL/6J-*Tyr^c-2J/^Tyr^c-2J^* mice, which have melanocytes but do not make melanin, were used as an additional source of meningeal tissue. All samples were positive for *Actin*. As expected, the B16 melanoma cell line, as well as the meninges from C57BL/6J and C57BL/6J-*Tyr^c-2J/^Tyr^c-2J^* mice, were positive for *Mitf-M* and *Pmel* (Figure 4A). However, RT-PCR performed on C57BL/6J-*MITF^mi−vga9^/Mitf^mi−vga9^* mice showed absence of both *Mitf* and *Pmel* transcripts (Figure 4A). Therefore the presence of melanocyte specific *Mitf-M* and its downstream target *Pmel* further suggests the presence of bona fide melanocytes in the meninges isolated from the mouse olfactory bulb.

### The Pigmented Cells are Melanocytes

**Figure 5:**
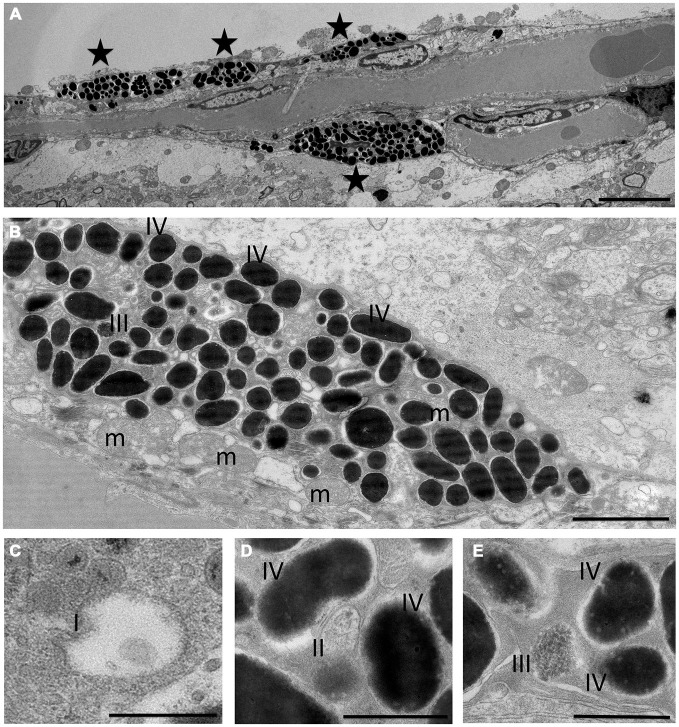
**Transmission electron microscopy of pigment cells.** Morphology of melanocytes **(A,B)** and melanosomes **(C–E)** in olfactory bulb at the electron microscope level. Asterisks in **(A)** denote melanocytes or dendrites of melanocytes. Stage I (I), stage II (II) and stage III melanosomes (III) can be seen between a large number of stage IV melanosomes **(B–E)**. m: mitochondria. The scale bar represents 5 μm in **(A)**, 2 μm in **(B)** and 500 nm in **(C–E)**.

The pigment containing cells have stellate morphology, they are dendritic and depend on Mitf, all typical characteristics of melanocytes. The mammalian brain coverings consist of three layers of protective tissue, namely the dense collgenous dura, the arachnoid and the pia, the latter two collectively called the leptomeninges. In order to determine in which cell layer the pigmented cells are located, we utilized classical histochemical staining techniques to stain tissues located between the olfactory bulb and cerebral cortex and around the pterygopalatine artery. Both the hematoxylin-eosin (H&E) and Masson-Hamperl (specific for melanin) stains showed darkly pigmented cells located within the leptomeninges between the olfactory bulb and cerebral cortex and surrounding the pterygopalatine artery (Figures 4B,C). To characterize these cells further, we used TEM to determine whether they contain melanosomes of all stages and had not simply phagocytosed extracellular melanin. Pigment cells from both the olfactory region and the pterygopalatine region were examined. Examination with TEM revealed that in both regions the pigmented cells contained melanosomes of all four stages of development (Figure 5).

## Discussion

Here, we describe the location of pigmented, dendritic cells in the brain coverings of the mouse as well as proximal to the optic nerve. These cells are found in six distinct areas of the meninges in C57BL/6J mice, they contain all stages of melanosomes and they express *Mitf*. They are not visible in albino mice suggesting that their pigment depends on tyrosinase. Also, they are not found in homozygous *Mitf^mi−vga9^* mice, demonstrating that the presence of these cells depends on this master regulator of melanocyte development and differentiation. Interestingly, fewer pigmented cells were observed in *Mitf^mi−vga9^* heterozygotes suggesting that these cells are sensitive to the loss of *Mitf* activity. These observations strongly suggest that these pigmented cells in the meninges are melanocytes. Only one of the areas we showed to contain melanocytes, the area between the olfactory bulb and the cortex, has previously been described to contain pigment cells in the mouse (Markert and Silvers, 1956; Barden and Levine, 1983). Barden and Levine (1983) reported the distribution of pigmented cells in the rat similar to that illustrated here. Pigmented cells have also been described in the meninges of other mammals (Goldgeier et al., 1984; Morse and Cova, 1984; Fetissov et al., 1999). Their distribution seems to vary between species. However they are always to some extent associated with the meningeal vasculature. This location is consistent with the angiotropism observed for melanoma cells and may suggest that melanocytes can travel along this route (Lugassy et al., 2014). At present, we do not have an explanation for the variation in the presence of these cells in the different areas of the brain. Age appears to be a factor. Local variations in the presence of melanocytes have not been reported for other tissues. In mouse heart valves and septa the number of melanocytes has been shown to be related to that in the skin (Yajima and Larue, 2008). A similar relationship with skin pigmentation has been observed in meningeal melanocytes of Ugandan Africans (Lewis, 1969). More detailed analysis of melanocyte distribution in different tissues may reveal previously undetected variation.

Melanocytes are derived from neural crest cells, known for their multipotency and migration ability during development. Using *in situ* hybridization analysis, Baxter and Pavan (2003) showed cells expressing Pmel, a melanoblast marker, at the dorsal surface of the midbrain/hindbrain boundary and extending laterally and along the lateral surface of the forebrain/midbrain boundary during development. They showed that these cells depend on *Mitf*. Similar cranial neural crest cells have been described in chickens (Baker et al., 1997). The meningeal melanocytes described here are likely to arise from the cranial neural crest. Alternatively, they may be derived from neural crest derivatives such as Schwann cell precursors (Adameyko et al., 2012), olfactory ensheathing glia (Barraud et al., 2010) or from multipotent cells in the dura that also are of a neural crest origin (Gagan et al., 2007). Classical lineage tracing and analysis of the temporal distribution of melanocytes in the meninges can be used to establish this.

The role of meningeal melanocytes remains unknown. The anatomical locations of these melanocytes in areas susceptible to infection such as the olfactory bulb via the olfactory epithelium, might suggest a possible role for these cells in the immune response. This is consistent with previous observations that melanocytes produce proinflammatory cytokines such as IL-8 (Miniati et al., 2014) and may modulate inflammation by NO production (reviewed in Tsatmali et al., 2002). NO also plays a key role in headaches, such as migraine, through its vasodilation effect on the meningeal vasculature. In that light it is interesting to note that meningeal pigment cells have recently been linked with aneurysm formation (Schulter et al., 2014) suggesting that these cells may play a role in the vasculature.

Diseases with known connections with melanocytes can roughly be grouped into neoplastic diseases and diseases of possible autoimmune etiology such as Vogt-Koyanagi-Harada syndrome (Greco et al., 2013). The diseases that most clearly involve meningeal melanocytes are the primary meningeal melanocytic neoplasms and their variants. Primary meningeal melanocytic neoplasms are rare potentially lethal forms of cancer in humans. According to the WHO guidelines for classification of tumors of the central nervous system (Louis et al., 2007) they are classified as diffuse meningeal melanocytosis, meningeal melanocytoma, malignant meningeal melanoma and meningeal melanomatosis. The most common is meningeal melanocytoma, occurring in 1 in 10 million individuals (Liubinas et al., 2010). Meningeal melanocytoma is a generally benign neoplasm, which can undergo malignant transformation (Brunsvig et al., 2011; Wang et al., 2011). A common site for a melanocytoma is in Meckels cave (Botticelli et al., 1983; Chen et al., 1997; Leonardi et al., 1998; Kurita et al., 2000; Tregnago et al., 2015) which contains the trigeminal ganglion and lies adjacent to the internal carotid artery (Arslan et al., 2012). This corresponds to the pterygopalatine artery in our observations as it arises from the internal carotid in rodents. This suggests that these neoplasms might originate in resident melanocytes in the meninges. Pedersen et al. (2013) created a mouse model for meningeal melanoma by expressing oncogenic NRAS^G12D^ in melanocytes of developing embryos. Although this mutation did not induce cutaneous melanoma, it resulted in early-onset primary melanoma of the CNS. The disease was most commonly located in the leptomeninges surrounding the anterior portion of the CNS, most notably around the olfactory bulb. Based on our results it is likely that these melanomas arise from pre-existing melanocytes in these areas.

## Funding

This work was supported by grant 120405021 from the Icelandic Research Fund to ES and PHP. ES and LL were supported by PHC JULES VERNE 2014 (31891VM). LL and FG were supported by Ligue Nationale Contre le Cancer (Equipe labellisée), Cancéropole Ile-de-France, INCa and Pair Mélanome. IH and GR were supported by the French National Research Agency through the “Investments for the Future” program (France-BioImaging, ANR-10-INSB-04). LL, FG, IH and GR acknowledge the PICT-IBiSA, member of the France-BioImaging national research infrastructure, supported by the CelTisPhyBio Labex (N ANR-10-LBX-0038) part of the IDEX PSL (N ANR-10-IDEX-0001-02 PSL).

## Conflict of Interest Statement

The authors declare that the research was conducted in the absence of any commercial or financial relationships that could be construed as a potential conflict of interest.
